# Role of Plastid Transglutaminase in LHCII Polyamination and Thylakoid Electron and Proton Flow

**DOI:** 10.1371/journal.pone.0041979

**Published:** 2012-07-27

**Authors:** Nikolaos E. Ioannidis, Oriol Lopera, Mireya Santos, Josep M. Torné, Kiriakos Kotzabasis

**Affiliations:** 1 Department of Biology, University of Crete, Voutes University Campus, Heraklion, Crete, Greece; 2 Departament de Genètica Molecular, Centre for Research in Agricultural Genomics, CRAG (CSIC-IRTA-UAB-UB), Barcelona, Spain; University of California - Davis, United States of America

## Abstract

Transglutaminases function as biological glues in animal cells, plant cells and microbes. In energy producing organelles such as chloroplasts the presence of transglutaminases was recently confirmed. Furthermore, a plastidial transglutaminase has been cloned from maize and the first plants overexpressing *tgz* are available (*Nicotiana tabacum* TGZ OE). Our hypothesis is that the overexpression of plastidal transglutaminase will alter photosynthesis via increased polyamination of the antenna of photosystem II. We have used standard analytical tools to separate the antenna from photosystem II in wild type and modified plants, 6 specific antibodies against LHCbs to confirm their presence and sensitive HPLC method to quantify the polyamination level of these proteins. We report that bound spermidine and spermine were significantly increased (∼80%) in overexpressors. Moreover, we used recent advances in *in vivo* probing to study simultaneously the proton and electron circuit of thylakoids. Under physiological conditions overexpressors show a 3-fold higher sensitivity of the antenna down regulation loop (qE) to the elicitor (luminal protons) which is estimated as the ΔpH component of thylakoidal proton motive force. In addition, photosystem (hyper-PSIIα) with an exceptionally high antenna (large absorption cross section), accumulate in transglutaminase over expressers doubling the rate constant of light energy utilization (Kα) and promoting thylakoid membrane stacking. Polyamination of antenna proteins is a previously unrecognized mechanism for the modulation of the size (antenna absorption cross section) and sensitivity of photosystem II to down regulation. Future research will reveal which peptides and which residues of the antenna are responsible for such effects.

## Introduction

Photosynthesis is one of the most important biological process in cellular physiology producing energy (in terms of ATP), oxygen and reducing power (in terms of NADPH_2_). Higher plants perform photosynthesis in special sites, the thylakoids that house the main photosynthetic subcomplexes (PSII, PSI, cytb_6_f and ATPase). Two main routes of electrons are operating in thylakoids, the so called linear electron flow (LEF) involving PSII, cytb_6_f and PSI and the cyclic electron flow (CEF), where only PSI and cytb_6_f are engaged. Both processes (LEF and CEF) release protons in lumen (the interior of thylakoids) and thus equilibrium is established between the efflux of protons through the ATPases and the formation of ATP from ADP and P_i_ in the stroma exposed part of the enzyme. The proton motive force (*pmf*) has two components, one osmotic termed (ΔpH) and one electric (Δψ) equally competent in ATP synthesis [1]. *Pmf* in thylakoids has an additional protective role, which is equally important for the photosynthetic process [2]. More particularly, photosynthesis is being on the short-term automatically regulated (with no direct need for de novo synthesis of proteins or other DNA/RNA related regulatory steps) at the level of light harvesting. Key role in the autoregulation of photosynthesis plays the so called qE response (activated in a few seconds). This is a switch of antenna of PSII from an efficient light harvesting and exciton migration mode to an energy dissipative mode, which is gradually more and more activated in accordance to the need for photoprotection. The photoprotective loop is activated by the ΔpH component of *pmf*, while zeaxanthin formation via xanthophyll cycle in thylakoids is believed to increase the efficiency of energy dissipation [3]–[5].

Rather overlooked factors of the thylakoid system are transglutaminases (TGases). These enzymes catalyze post-translational modification of proteins by establishing ε-(γ-glutamyl) links and covalent conjugation of polyamines [6]. These wide spread enzymes are considered as biological glues (one of the most known is the Factor XIII human blood coagulation factor) that crosslink proteins via covalent bonds. In plants, using *Helianthus tuberosus* isolated leaf chloroplasts, it was shown that some antenna proteins of the photosystems (LHCII, CP29, CP26 and CP24) were substrates of TGase activity [7]. By means of an animal anti-TGase antibody, a TGase was immunodetected as a unique 58 kDa band, starting from protein extracts of maize meristematic calli and their isolated chloroplasts. Furthermore, the detected TGase activity was shown to be light sensitive, affected by hormone deprivation and with a light/dark rhythm [8], [9]. Studies of immunogold localization of transglutaminase in different maize cell types using a plant TGase antibody from *Helianthus tuberosus,* showed that the enzyme is specifically localized in the chloroplast grana-appressed thylakoids, close to LHCII, its abundance depending on the degree of grana development [10]–[12]. An important step in elucidating the role of plastidal TGase was the isolation for the first time in plants of two related complementary maize cDNA clones, *tgz15* and *tgz21*, encoding active maize TGase (TGZ) [13], [14]. Their expression was dependent on length of light exposure, indicating a role in adaptation to different light environmental conditions, including natural habitats [15]. Proteomic studies indicates that maize chloroplastic TGase is a peripheral thylakoid protein forming part of a specific PSII protein complex which includes LHCII, ATPase and PsbS proteins [16]. The heterologous expression of TGZ in *E. coli* cells indicates that the native substrate preferences in the recombinant TGZp were maintained and also that the enzyme activity was light dependent only in the case when light-grown plant protein substrates were used [17], [18]. Taking into account all the described results, it has been hypothesized that TGases are implicated in the photosynthetic process [14], [15], [19]. First evidence for a role of plastidial TGase in the thylakoids 3D architecture comes from tobacco over expressing maize TGase (TGZ) [20]. Transformed tobacco chloroplasts, with a 4-fold increase of TGase activity, showed stroma thylakoid depletion and granum size increase. At the same time, as a consequence of increased TGZ activity, thylakoid-associated polyamine content also increased [20].

Polyamines (PAs) are low molecular weight aliphatic amines that are almost fully protonated under normal pH values, so having a net charge of up to +4. The main polyamines, putrescine (Put), spermidine (Spd) and spermine (Spm) are normally found associated to LHCII antenna proteins of higher plants [21]. As indicated earlier, a covalent polyamine attach to thylakoid proteins, specifically LHCII, CP29, CP26 and CP24, has been demonstrated as a consequence of plastidial TGase activity [7]. Recently, it was also shown that a plastidial TGase activity in maize (*Zea mays L*.) polyaminylates LHCII in a light dependent way, although it is discussed whether this light sensitivity is due to the enzyme or to the substrate [22]. In that work, the two terminal amino-groups of PAs conjugate to one or two glutamine residues giving rise either to mono-(γ-glutamyl)-PAs (mono-PAs) or bis-(γ-glutamyl)-PAs (bis-PAs). These authors discuss if the additional positive charges inserted on proteins by the protein-bound PAs may induce conformational changes or even affect stacking.

Besides the bound form of polyamines found in LHCII, free forms have also key roles in photosynthesis. Free putrescine was shown to increase photophosphorylation by 70% in isolated thylakoids [23], and by 40% the Δψ component of thylakoidal *pmf* in intact tobacco leaves [24]. More recently, Spd and Spm were shown to induce quenching in isolated LHCII from green algae and higher plants mimicking the effects of known stimulators of qE such as dibucaine [25], [26]. Polyamines affect significantly the functionality of key photosynthetic complexes (reviewed in [27]) and recently become apparent that polyamines are promising factors in photosynthesis research.

Ideally, a plant with a high level of LHCII polyamination *in vivo* could provide an excellent tool towards the elucidation of the physiological role of this post translational modification of the PSII antenna. In this work we ask whether antenna of PSII (Lhcb1–6) has a different polyamination status due to the higher TGase activity in tobacco plants that over express plastidic TGZ. Then, we are interested in the consequences with respect to antenna regulation under this polyamination status. Due to the key role of LHCII in photosynthesis processes, we present also the behavior of several photosynthetic parameters measured in intact *tgz*-transplastomic plants, exploring recent advances in *in vivo* spectroscopy. Here we show that Spd and Spm content in the isolated LHCII is increased by about 80% due to TGZ over-expression, and that in vivo PSII antenna regulation of the over-expressers is about four times more sensitive to the ΔpH component of *pmf* in comparison to the WT. Furthermore, an in-depth study of the *in vivo* proton and electron circuit of these grana-rich plants is presented. A previously unrecognized mechanism of light harvesting modulation via transglutaminase attachment of polyamines is emerging.

## Results

### Responses of Photosynthetic Electron Transport and Photoprotection

**Figure 1:**
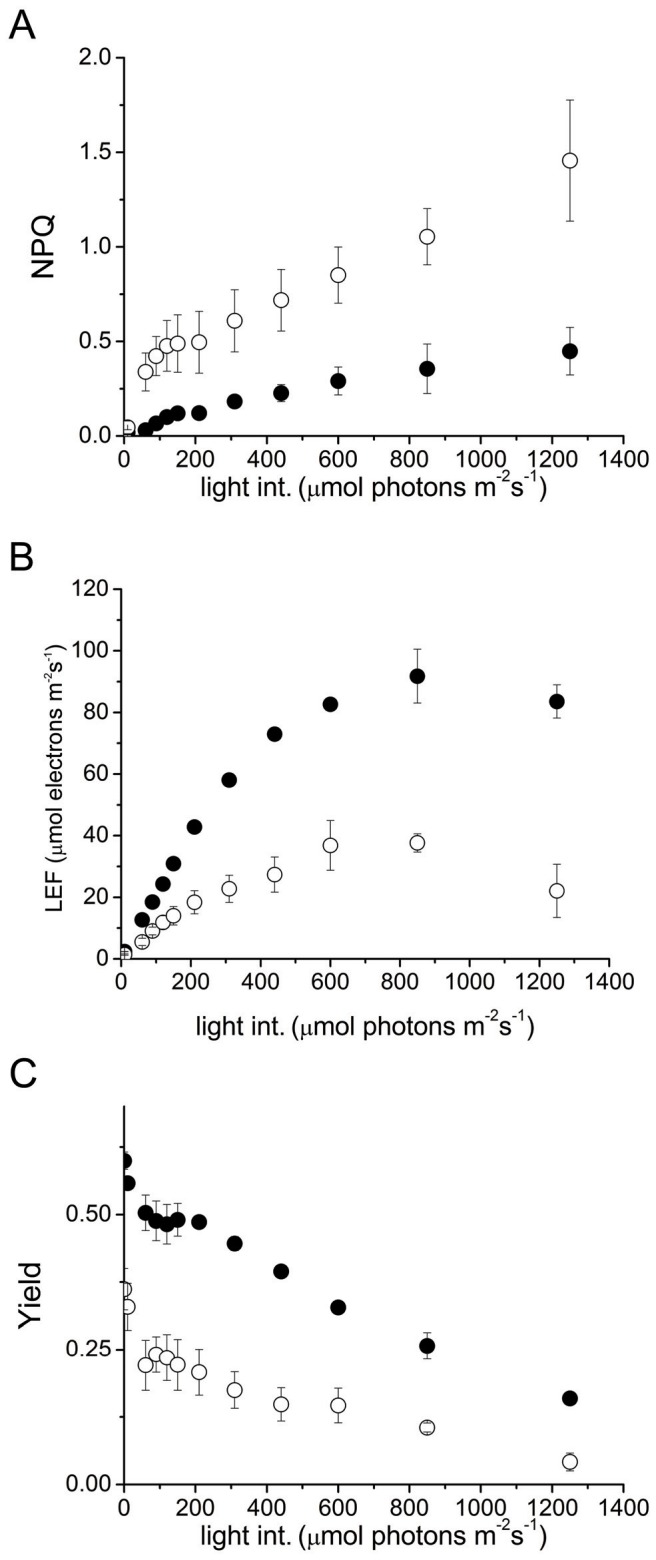
PAM Fluorimetry in leaves. Rapid light curves of basic photosynthetic parameters for wild type (closed symbols) and overexpressors of TGZ (open symbols). Data are presented as the means ± SE (n = 3). (A) Non photochemical quenching (NPQ) is higher for TGZ overexpressors over a range of light intensities. (B) Linear electron transport (LEF) is lower for TGZ overexpressors and saturates at around 600 µmol photons m^−2^s^−1^. (C) Yield of the overexpressors is only half of that of WT over the range of light intensities tested.

Overexpression (OE) of TGZ in tobacco significantly alters the light curves of NPQ, LEF and Yield (Figure 1). NPQ activation is abruptly and highly induced even in low light conditions in TGZ OE. At higher light conditions, NPQ in transformed tobacco is about 3-fold higher than in WT. LEF saturates at about 600 µmol photons m^−2^s^−1^ in TGZ OE and at around 800 µmol photons m^−2^s^−1^ in WT. In absolute values, maximal LEF is about half of the WT values. Similarly, yield in TGZ OE is only half of the yield in WT for the range 0–1200 µmol photons m^−2^s^−1^. These plots derive from quite short in duration protocols and as the light intensity is progressively getting higher they reflect differences in the photosynthetic reflexes of the two lines. Additional information about photosynthesis one could gain from steady state conditions.

**Figure 2:**
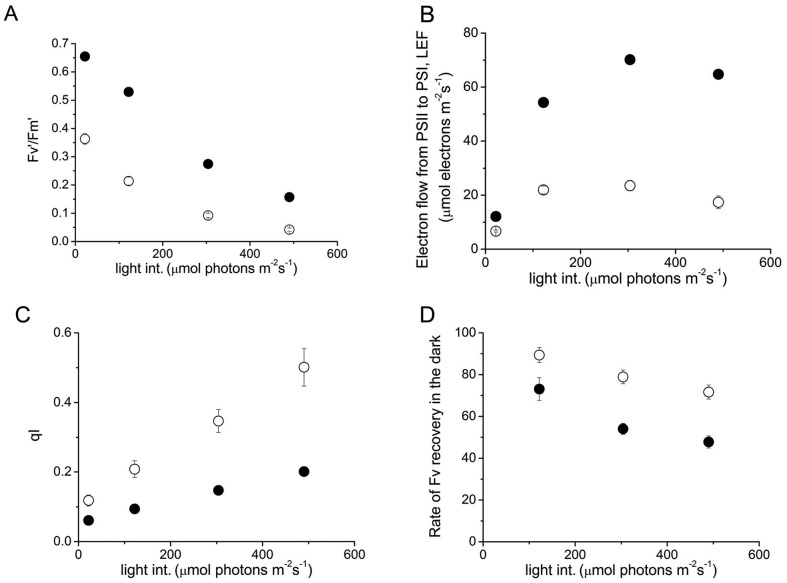
Steady state photosynthesis in leaves. In vivo fluorescence spectroscopy for intact WT (closed symbols) and TGZ overexpressors (open symbols). Data are presented as the means ± SE (n = 8). (A) Effective quantum yield at steady state (Fv/Fm’). (B) linear electron flow at steady state (LEF). (C) photoinhibition quenching (qI). (D) rate of Fv recovery in the dark.

Extending to 10 min the time of actinic illumination, linear electron flow (LEF) (Figure 2A) and effective quantum yield of photochemical energy conversion in PSII (Fv/Fm’) (Figure 2B) were measured. As can be seen, steady state LEF and Fv/Fm’ values were less than half of the WT values. In addition, qI was higher in TGZ OE than in WT (Figure 2C), i.e.: qI at 500 µmol photons m^−2^s^−1^ was about 0.5 in TGZ OE (a relatively high value), and about 0.2 for WT. Interestingly, the fluorescence recovery in the dark, as estimated by the Fv values, shows significant faster relaxation for TGZ OE in comparison to the WT (Figure 2D).

### Comparison of the Photosynthetic Electron and Proton Circuits in WT and TGZ OE

**Figure 3:**
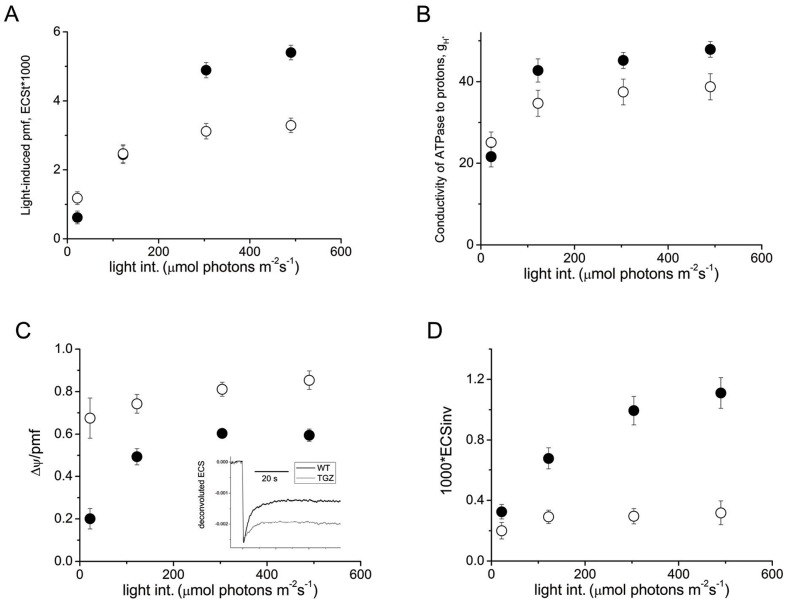
Estimates of the protonic circuit of photosynthesis. In vivo monitoning of the protonic circuit of photosynthesis in WT (closed symbols) and TGZ OE (open symbols). Data are presented as the means ± SE (n = 8). (A) Light-induced pmf as estimated by the ECS_t_ parameter. (B) Apparent conductivity of the ATPase of thylakoids (gH+). (C) The electric component of pmf (Δψ/*pmf*) as estimated by three wavelength deconvolution (insert shows typical traces). (D) Estimates of the ΔpH component of *pmf* in thylakoids as estimated by the ECS_inv_ parameter.

**Figure 4:**
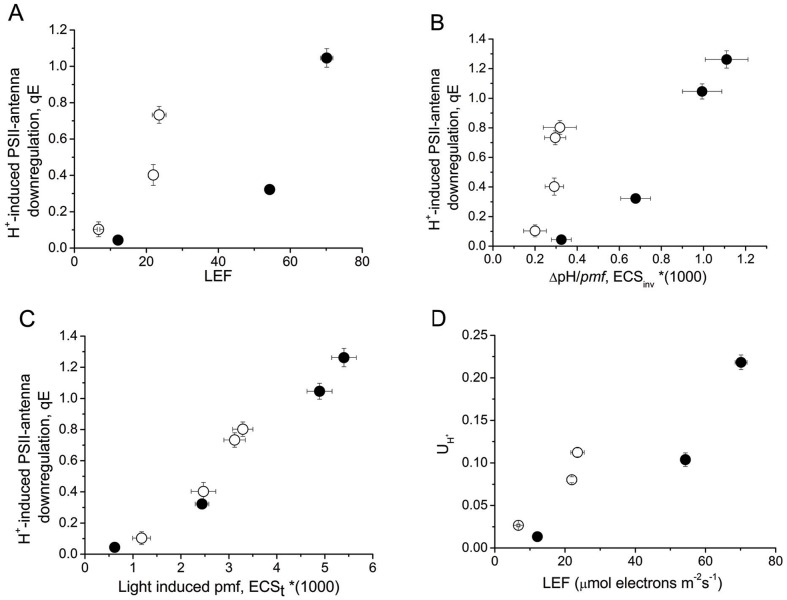
Regulation of the protonic circuit of photosynthesis. WT (closed symbols) and TGZ OE (open symbols). Data are presented as the means ± SE (n = 8). (A) Sensitivity of antenna downregulation (qE) to LEF. It is apparent that for the same LEF TGZ OE shows a higher qE response. (B) Sensitivity of qE to the ΔpH component of *pmf*. This increased sensitivity indicates that antenna properties are significantly different from WT. (C) Sensitivity of qE to light induced pmf follows in transformed tobacco the same trend as in WT. (D) Proton efflux in thylakoids of TGZ OE is getting more sensitive to LEF in comparison to WT.

According to the method of ref [28], *in vivo* monitoring of the protonic circuit of photosynthesis is feasible. Light induced *pmf* is estimated as the amplitude of dark induced relaxation kinetics of the 520 nm signal [28]. Applying this methodology, in low light conditions light-induced *pmf* was about 93% higher in TGZ OE, but at higher light conditions TGZ OE showed only 60% of the WT value (Figure 3A). A similar trend holds for the apparent conductivity of ATPase to protons (gH^+^). The gH^+^ values at 22 µmol photons m^−2^s^−1^ were a little higher for TGZ OE (∼17%), but they were getting lower (∼20%) at higher light conditions (up to about 500 µmol photons m^−2^s^−1^), always with respect to WT (Figure 3B). Partitioning of *pmf* in the two components presented striking differences. TGZ OE stored their *pmf* mainly as electric field with a value of Δψ/*pmf* of around 0.8 and only the remaining 20% as ΔpH (Figure 3C). WT showed a Δψ/*pmf* of around 0.6 at 500 µmol photons m^−2^s^−1^. Typical deconvoluted traces used for the determination of Δψ/*pmf* are shown as an insert in Figure 3C. The low ΔpH was also reflected in the values of ECS_inv_ that were much smaller in the case of TGZ OE (Figure 3D). TGZ OE showed larger sensitivity of antenna downregulation qE to the LEF (Figure 4A). At around 20 µmol photons m^−2^s^−1^, qE was only marginally induced in WT, but it presented relatively high values (about 0.75) in TGZ OE. The sensitivity of the PSII antenna to the ΔpH component of *pmf* was also greater in TGZ OE (slope of the linear fitting curve is 5.628) than in WT (slope 1.603). It seems that only a small ΔpH difference, as probed by the ECS_inv_ parameter, was adequate to activate to a large extent the qE response (Figure 4B). On the contrary, antenna regulation as a function of light induced *pmf* seemed marginally affected and only at higher *pmf* values there was, if any, a deviation from WT curve (Figure 4C). In addition, proton efflux from thylakoids, *ν*_H+,_ was significantly more sensitive to LEF in TGZ OE than in WT (Figure 4D). High proton effluxes under low LEF in TGZ OE may originate from higher activation of cyclic electron flow around PSI.

### Photosystem Architecture, Functionality and Antenna Organization in WT and TGZ OE

**Figure 5:**
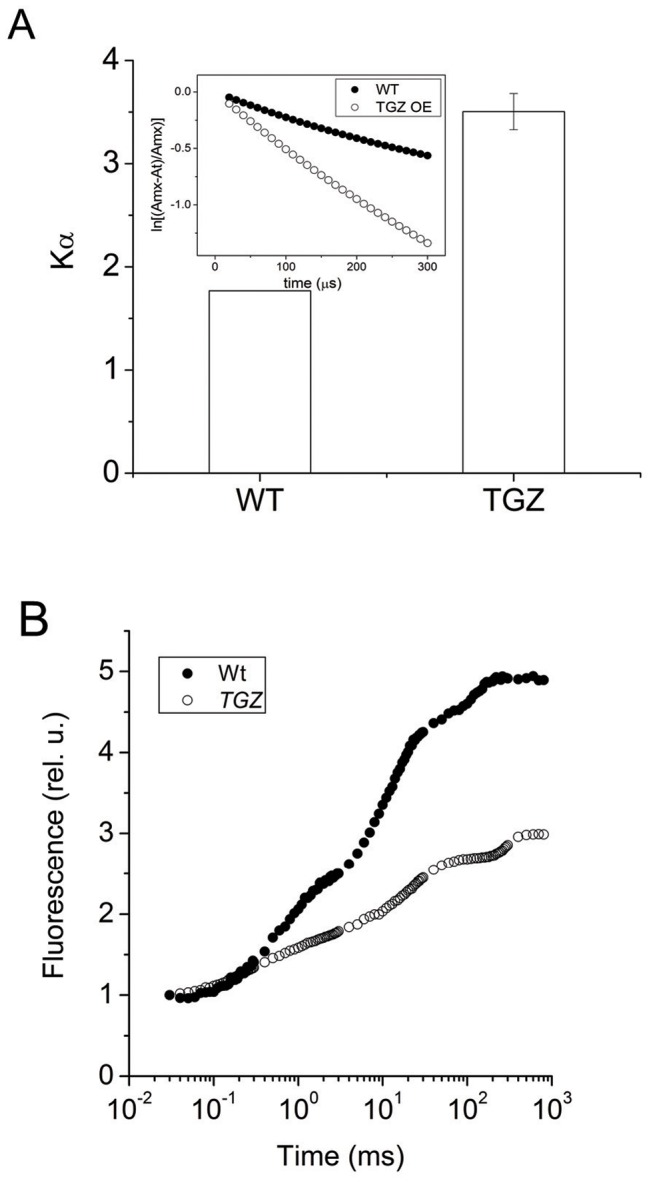
Non invasive probing of tobacco antenna by rapid fluorescence induction curves. (A) TGZ overexpression induces an increase Ka of PSIIa centers. (B) TGZ overexpession significantly affects the shape of the OJIP curve (fluorescence induction curve).

According to the previously described grana ultrastructure [20], PSII units in TGZ OE showed an increase of the PSIIa centers (centers with large antenna or large absorption cross section) as a fraction of the total number of reaction centers [20]. The rate constant Kα of these centers is much higher than the WT indicating that photons are more efficiently harvested and utilized by the transformed tobacco plants (Figure 5A). This finding indicates that it is not only the fraction of PSIIα that increases, but also the absorption cross section of each PSIIα is doubling due to OE overexpression (we call these centers hyper-PSIIα centers). Fluorescence induction curves normalized to Fo, showed lower P values indicating that the maximum quantum yield (estimated as Fv/Fm) was lower in comparison to the WT (Figure 5B).

### PSII Antenna Bound Polyamine Content in WT and TGZ OE

**Figure 6:**
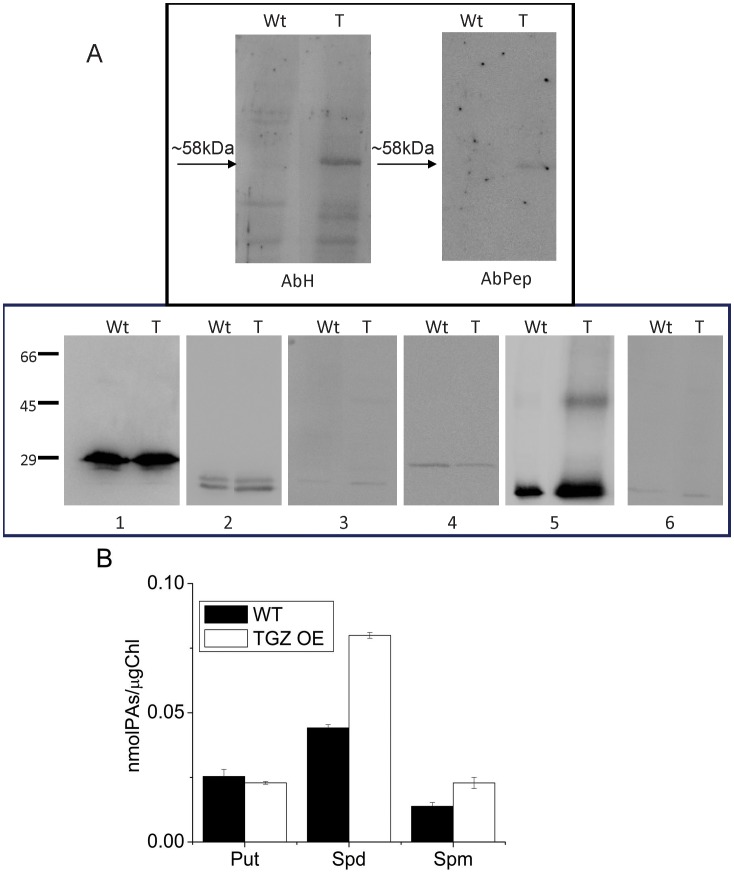
Changes in antenna properties between WT and TGZ and the polyamines bound in LHCII, CP29, CP26 and CP24. (A) Western blot immunolocalization indicating that the fraction used for polyamine determination contains TGZ in the OE, and Lhcb1–6 antenna proteins in WT and in TGZ OE. AbH (1∶1000) and Abpep (1∶500), antibodies against generic TGase and against TGZ, were respectively used. 1 to 6, anti Lhcb1–6 antibodies (1∶5000), were respectively used. (B) HPLC determination of bound-polyamines of the same fraction as above, indicating that TGZ over-expression increases bound Spd and Spm in the antenna of PSII.

Taking into account that during *in vivo* photosynthesis, antenna regulation of TGZ OE was more sensitive to ΔpH component of *pmf* than the WT line, it seemed interesting to determine the level of polyamination of PSII antenna in the two lines. After isolating the LHCII antenna of PSII by sucrose gradient ultracentrifugation of solubilized thylakoids, we confirmed by western blot analysis, that the band used for polyamine analysis presented all major LHCII proteins in both lines, as well as TGZ protein in the case of OE, (Figure 6). As can be seen in Fig. 6A (above), the presence of TGZ in the OE fraction was corroborated by the two used antibodies. A clear band at approximately 58 kDa in the TGZ OE and other less-concentrated protein bands in the WT that might correspond to endogenous tobacco TGase or to non-specific signals, were detected when AbH (a generic plant-TGase antibody, [10]) was used. However, when an antibody against a specific TGZ C-terminal sequence was used (AbPep) [16], a unique 58 kDa band (a MW corresponding to TGZ) was obtained in the OE and not in the WT. With respect to the presence of Lhcb1–6 proteins in the same band, (Fig. 6A, bellow), the only difference observed was in the case of Lhcb5 (CP26), that presented a clear signal at 58 kDa in the TGZ OE extract that is not present in the WT extract. This protein band might correspond either to a dimerization of Lhcb5 due to TGZ over-expression and/or to a co-migration of TGZ with this Lhcb dimer. Protein-bound polyamine determination (Fig. 6B) revealed striking differences in the profile of transformed tobacco extracts with respect to the WT. Bound Spd and Spm were increased about 80% in TGZ OE, while bound Put was less affected.

### Ultrastructure Changes Due to TGZ Over-expression

**Figure 7:**
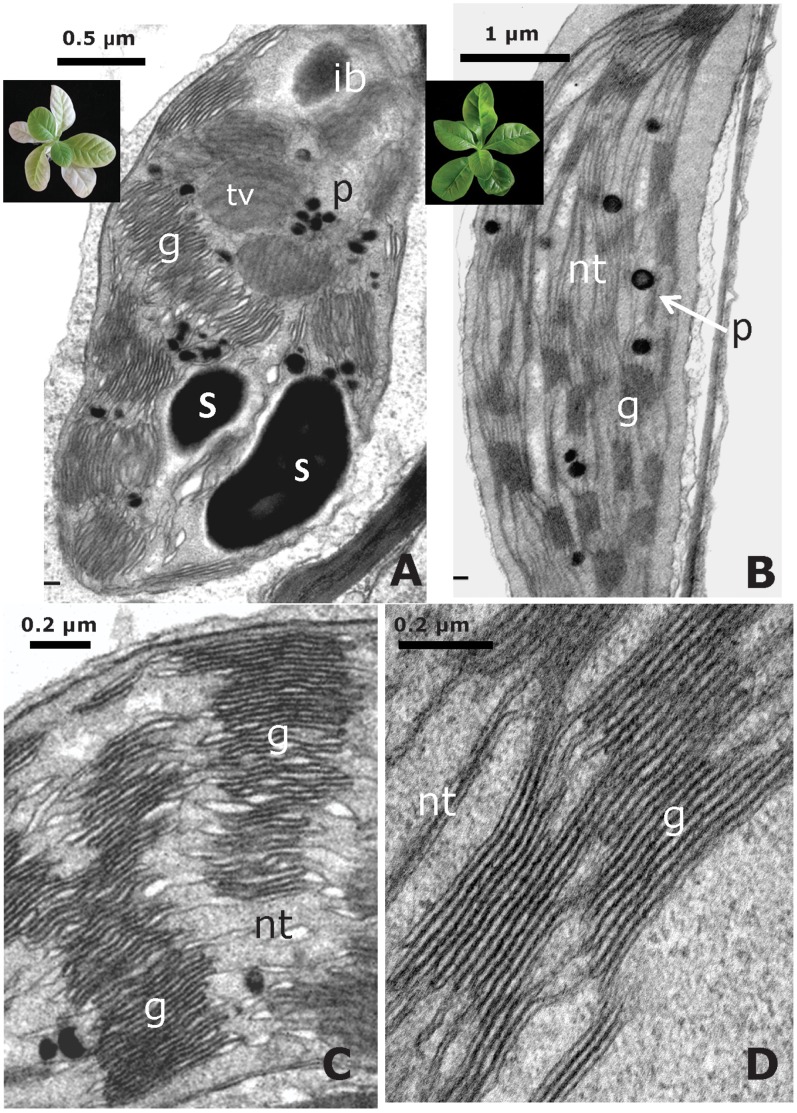
Chloroplast ultrastructure of WT and TGZ over-expressing *Nicotiana tabacum* leaf cells. The TGZ-transformed chloroplasts (A,C) show an increased grana appresion and a reduced stroma thylakoid network versus the WT (B,D). In the TGZ over-expressing chloroplasts, large grana with an increased number of appresed thylakoids and a decline in thylakoidal interconections are observed. Flattened thylakoids and starch grains can be also detected sometimes in the TGZ-transformed chloroplasts (C). According to the TGZ chloroplast age, inclusion bodies of different size were visible (A). In the WT chloroplast (B,D), the thylakoid architecture is normal. Insert Fig. 7A: aspect of an *in vitro-*grown TGZ-transformed plant showing the youngest green leaves and the oldest white leaves. Insert Fig. 7B: aspect of an *in vitro-*grown WT plant. **ib** inclusion body, **g** grana, **nt** non-apressed thylakoid, **p** plastoglobuli, **S** starch grains, **tv** top views of grana.

In our previous works [20], [29], [30] we have shown that *in vitro* culture not altered the architecture of thylakoid network in WT plants, where normal grana and stroma thylakoids can be observed. However, TGZ over-expression had striking effects in the thylakoid network, showing significantly more stacked membranes per granum than in the WT (40 to 60 layers in the over-expressers, with respect to 15 to 20 in the WT), and a significant decrease of thylakoidal interconnections between grana (see references above mentioned). Our recent electron microscopy data confirm that the striking trait of high stacking and big grana [20] is stable in these OE lines after many rounds of sub cultivation in vitro (figure 7A and C). At this point, we should underline that TGZ overexpression increase leaf hereterogenity with age, ranging from young-green leaves to old-white leaves (see insert figure 7A), thus, we had to cope with this issue. The results in the present work are always taken from green leaves (both for TGZ and WT) that were of high Fv/Fm and by analyzing data from leaves that had positive LEF values not only at the beginning but also at the end of the experiments.

### Comparison of Counter Ions and qE Response in WT and TGZ OE

**Figure 8:**
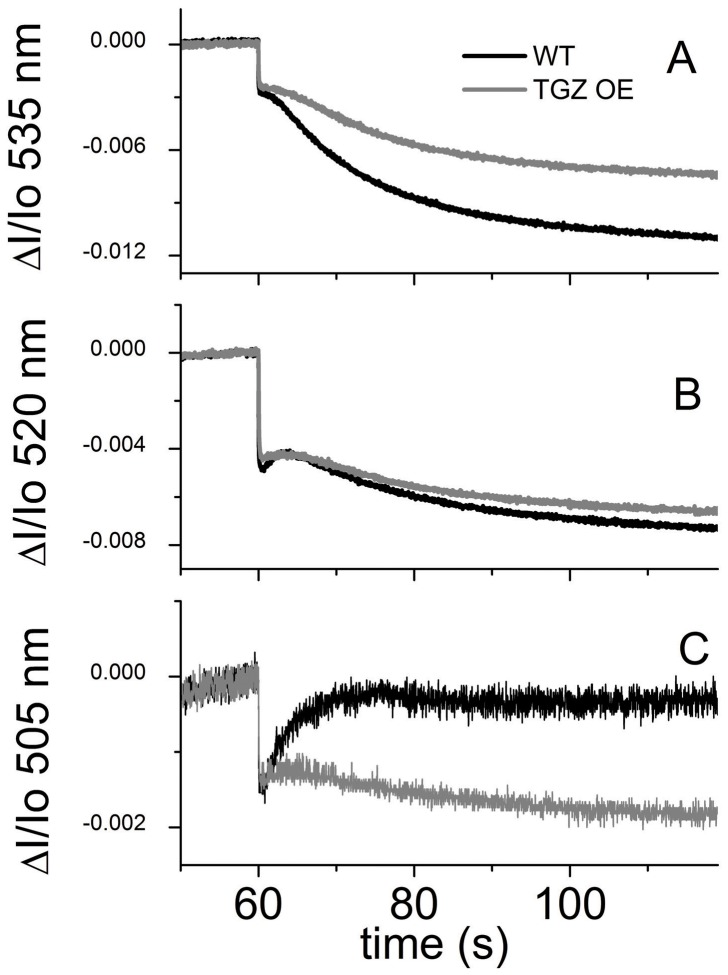
Absorbance spectroscopy in leaves. Comparison of the raw signals between WT (black) and TGZ OE (gray). (A) The signal at 535 nm that is rich is information related to electrochromic shift and to the qE effect (B) The signal at 520 that is rich mainly in electrochromic shift (C) The raw 505 nm is rich in information related to electrochromic shift but is less contaminated by the qE effect.

Actinic light triggers vectorial proton release in lumen, which in turn changes the ion fluxes between stroma and lumen. Upon shuttering of actinic light, proton release stops and ions are moving to find a new equilibrium. In Figure 8, we present the effect of shuttering of actinic light at the absorbance at 505 nm in TGZ OE and WT plants. The rapid drop at the first second corresponds to proton efflux, while the inverted peak after 10 more seconds should reflect counter ion movements. It is evident that TGZ OE showed only marginal counter ion movements (small peak after sudden drop) with respect to WT, although proton efflux is of similar extent to that in WT (please compare amplitude of sudden drop between WT and TGZ OE).

### Xanthophyll Cycle *in vivo* and Total Leaf PA Titer

**Figure 9:**
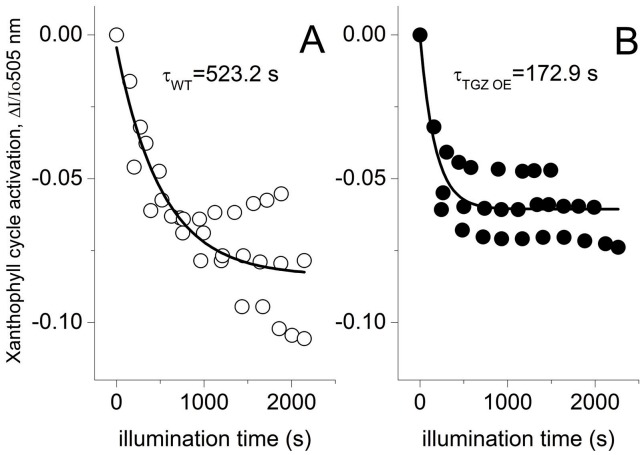
Activation of xanthophyll cycle in WT and TGZ OE. First order decay kinetics were fitted to data from 3 independent experiments and corresponding tau values are illustrated in the graphs. Plants were illuminated with 500 µmol photons m^−2^s^−1^ for about 30 min. (A) Activation of xanthophyll cycle in WT. (B) Activation of xanthophyll cycle in TGZ OE. In TGZ OE xanthophyll cycle is induced about 3 times faster. Upon shuttering actinic the signal at 505 nm was marginally changed after 10 min indicating that contamination of the 505 nm from the qE response peaking at 535–540 nm is low.

**Figure 10:**
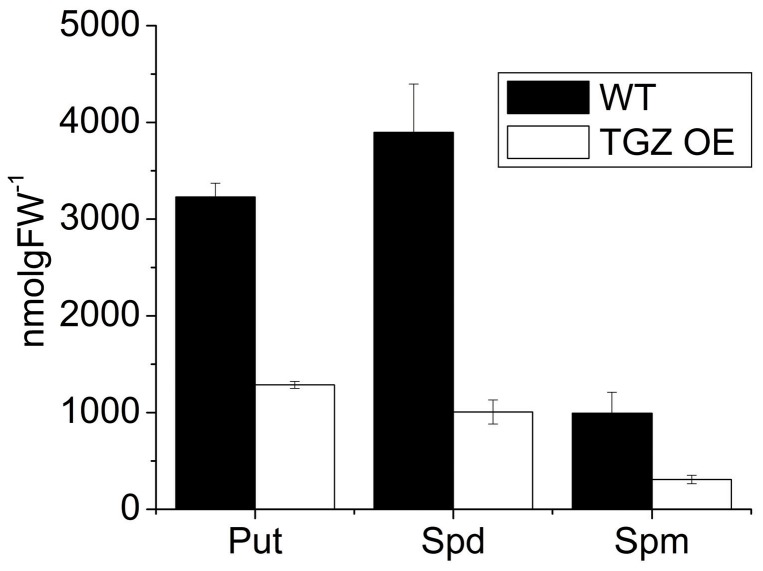
Polyamine titer in whole leaves. TGZ OE have three times less polyamines than WT. In TGZ OE, Put is 40%, Spd 26% and Spm 31% of the WT values. Data are presented as the means ± SE (n = 3).

The activation of xanthophyll cycle following the absorbance changes at 505 nm was estimated in both lines (Figure 9). As may be seen, zeaxanthin synthesis seems to have a τ of about 172 s in TGZ OE while τ was 523 s in WT. Significant differences were also detected in the titer of total leaf PAs between the two lines. TGZ OE accumulate 3 times less PAs in the leaf than WT (Figure 10).

## Discussion

Recently, we showed that thylakoid remodeling in tobacco chloroplasts is feasible through over-expression of maize plastidal transglutaminase in these plants [20]. Namely, TGase acts as a grana making enzyme (Figure 7). The mode of TGase action is related to the accumulation of PSIIa centers (centers with large antenna) [20]. Here we studied in more depth the effect of the increased activity of the plastidal transglutaminase and focus in PSII antenna polyamination status, in absorption cross section and efficiency of PSII and in a detailed functional characterization in vivo of the proton and electron circuit of photosynthesis both of which originate from PSII.

### Plants Overexpressing TGZ Show High Sensitivity of Antenna Down Regulation via qE

Here, we studied in depth the impact on curtain functional aspects of photosynthesis due to the increased TGase activity in the chloroplast. One of the most striking results of the present work is that TGase over expression makes antenna of PSII much more sensitive to down regulation via qE (Figure 4). Antenna of PSII is considered as the site of qE where photo protective energy dissipation takes place [31]. This type of quenching is sort of a holy grail for photosynthesis and thus any factor/enzyme that could improve our understanding regarding the mechanistic details is of particular importance.

### TGZ Overexpression Increases LHCII Bound Polyamines

**Figure 11:**
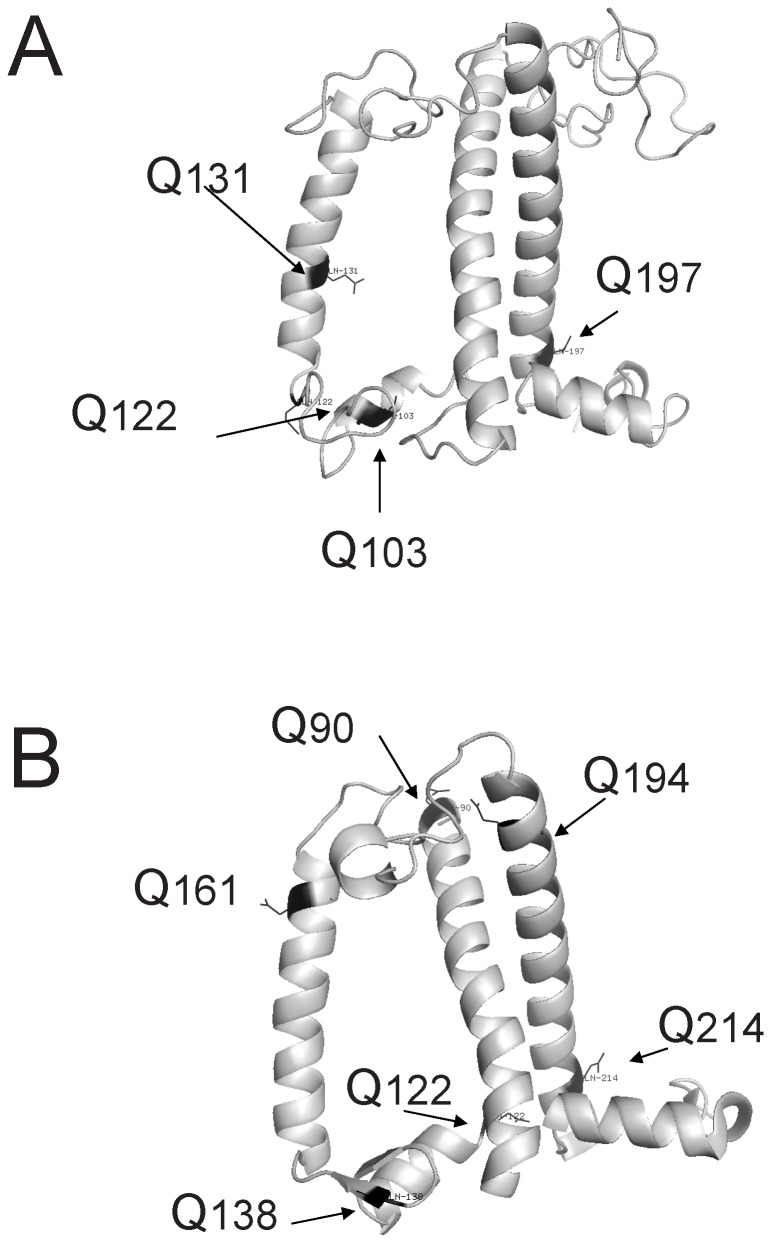
Model of antenna proteins from dicots. (A) Model of LHCII monomer from pea (from residue 10 to 232). Side view of LHCII showing the three transmembrane helixes (gray) and the glutamine residues (black) that could be potential substrate sites for the plastidal transglutaminase. Entry used for the model (*PDB ID: 2BWH*). (B) Model of CP29 from spinach (from residue 88 to 243). Side view of CP29 showing the three transmembrane helixes (gray) and the glutamine residues (black) that could be potential substrate sites for the plastidal transglutaminase. Entry used for the model (*PDB ID: 3PL9*). Please note that it is highly unlikely all these marked residues to be substrates for TGases. More likely substrates are those residues that are stroma exposed such as Q90 (panel B).

Recently, we showed that polyamines can induce NPQ *in vivo* [32] and in isolated LHCII [25], [26]. In addition, we showed that thylakoid associated polyamines and qE are increased in TGZ OE. In the present work, we showed that the amount of polyamines bound to LHCII is increased due to the increased activity of TGase. This finding confirms that LHCII is an *in vivo* TGZ substrate [7], [22] and that the impact of its post-translational modification is crucial for the photosynthetic apparatus [15], [16]. Co-isolation of substrate (i.e. LHCII) and enzyme (i.e. TGZ) in the same fraction of the sucrose gradient confirms and extends previous immunolocalization and proteomic studies showing that TGase is found in appressed regions of thylakoids [10] and isolated in a supercomplex, that once separated in a second dimension, included LHCII and PsbS proteins [16]. This supercomplex (reported in ref. [16]) could originate from co-migration rather than formation of a native complex. However, under our ultracentrifugation conditions co-existence of TGAse with LHCII in the same band indicates that a possible complex of the enzyme (i.e. TGase) and target protein (i.e. LHCbs) can not be excluded (Figure 6A). More particularly, thylakoid associated polyamines are naturally occurring amines in the LHCII of higher plants and algae [27], [33], [34]. Furthermore, polyamines of LHCII respond to environmental factors as if they were part of a regulatory mechanism that fine tunes the properties of PSII antenna [33]–[35]. Although polyamines can interact via hydrogen bonding and electrostatic interactions with the LHCII, more firm linkages via covalent attachment due to TGases are also well known (for a recent review see ref [19]). The present work indicates that polyamines bound to LHCII via TGases could increase the antenna size of PSII (absorption cross section) providing a reasonable explanation for the accumulation of PSIIa centers. Thus, a simple working model based on the findings of this work and the previous report [20] could be illustrated in Figure 11. PSII units with small antenna size recruit more LHCII proteins per reaction center and are transformed to PSII units with larger absorption cross section (PSIIa centers). Actually the antenna increase in PSIIα of TGZ OE is so large that Kα is two fold higher that in WT PSIIα centers. The higher antenna is plausibly the underlying cause of high qE sensitivity and high qI of the transformed tobacco. More particularly, increased photon harvesting of the TGZ OE due to bigger antenna size of PSII (in comparison to the WT) activates qE very early (i.e. even at relatively low light intensities). The higher sensitivity of qE to *pmf* does not imply that TGZ OE have higher capacity for qE. In other words maximal values of qE in TGZ are lower than maximal qE of WT (Figure 4C). Thus, there is a lower limit in light intensity that can be tolerated by TGZ OE in comparison to WT. At higher light intensities photoprotective qE is not enough and PSII is inactivated to a higher degree than WT. Thus qI at 500 µmol photons m^−2^s^−1^ is higher for TGZ OE than for WT plants (Fig. 2C).

### A Working Model for the Increase of PSII Centers with Big Antenna

The aforementioned increase of PSIIa could involve at least two different alternative mechanisms. One possibility is TGase to increase the amount of Spm and Spd in LHCII and this in turn to affect the binding affinity of LHCII to the PSII complex. Spd and Spm are positively charged at neutral pH and thus could alter the charge/conformation of LHCII and stimulate docking to PSII. Another possible mechanism is TGases to crosslink LHCII directly with the PSII complex via polyamine bridges (covalent bonding). Future studies could elucidate this important issue. Up-to-date, it is clear that LHCII trimers could bind strongly to PSII (S-trimers), or more lightly (M trimers), and that these complexes could form supercomplexes depending on light energy conditions [36], [37]. It is also cited that these phenomena are partially related to ΔpH, protonation and PsbS protein presence. In this respect, [38] working with *A. thaliana* EM and image analysis of thylakoid membrane fragments, observed that the frequence of semi-crystalline domains of photosystem II supercomplexes depends partially from the presence of PsbS protein, supporting the hypothesis that qE involves a PsbS re-organisation of PSII supercomplexes in the grana membrane. Nevertheless, this group agrees that other unknown factors, such as certain ions, may be also necessary for this bounding.

On the grounds that TGase crosslink glutamine residues with polyamine amino groups or with lysine residues, it is feasible to detect available docking sites in the primary structure of LHCb proteins via *in silico* analysis. Putative sites (Q residues) are marked in the model of Figure 11 based in the available 3D structure of LHCII (A) and CP29 (B). Moreover, based on the available primary structures of LHCII proteins, CP29 in tobacco has 11 glutamine residues (Q), while CP24 has 7, Lhcb1 has 5 and CP26 has 3 [39], [40]. It is highly unlikely all Q residues to be substrates of the plastidal transglutaminases. However, we illustrate all available information from structural data as way to put forward some considerations regarding their position, exposure to stroma enzymes and to their relative distance. These proteins (i.e. LHCII, CPs) contain many pigments and it is difficult for a stroma factor such as TGases to access residues close to lumen. More likely is TGase to modify Q residues that are more accessible (for example, Q90 and Q194 in CP29 seem good candidates either for mono-or for bis-conjugates). Assuming that some of the 26 available Qs are bound with a polyamine (e.g. Spermine) via mono conjugates, then the total charge of these 6 proteins can significantly increase. On the grounds that the optimal pH activity of the transglutaminase is from 6.5 to 8 [14] more probable target sites are the stroma exposed residues (e.g. Q90 in Figure 11B). This kind of modifications could be to some extent the origin of antenna heterogeneity reported by several groups in the past working either with proton domains within LHCII (Dilley group such as refs [41]–[44]) or with isoforms of LHCb that can not be explained only in terms of the *lhcb* gene variability [45]. Whoever is/are the docking residue(s) the advantage of TGase function seems to be important also under low light conditions where photons are rare energy source and LEF and *pmf* would operate better if light harvesting is optimal. In all cases, this “code” of transglutaminase-mediated LHCII modification, either by protein crosslinking (glutamine-polyamine-glutamine) or by polyamination (LHCII modification by Put, Spd or Spd) will be a subject of further research.

### Photosynthesis in Plants Overexpressing TGZ

Equally important are the results from the proton circuit of photosynthesis in TGZ OE. Light induced *pmf* is normally established in transformed tobacco for the light intensity range tested. Interestingly at low light (about 22 µmol photons m^−2^s^−1^) light induced *pmf* is about double in TGZ OE than in WT indicating that proton release is higher and/or proton efflux lower. According to ref. [46] either higher number of reaction centers and proton release or lower proton efflux through ATPases could be responsible for the increase in light induced *pmf*. The number of reaction centers per cross section is lower in TGZ OE because the chlorophyll content is less than half of the WT [20]. In addition, by comparing apparent conductivity of the CFoCF1 ATPase to protons we see that the decrease is small (about 15%) to account for the 70% higher *pmf*. Thus, we conclude that more efficient light harvesting due to the larger absorption cross section of the TGZ OE (as reflected also by the higher rate constant Kα) should be the cause of the higher light induced *pmf*. Perhaps as discussed above, plastidal TGAses provide the plant organism a system to increase PSII antenna size, so that more efficient photon absorption is feasible. Although these effects seem to operate in the long term (photoadaptation of PSII in low light conditions) a short term role could also be possible. Interestingly, it is known that Ca^+2^, GTP and light are factors that regulate the endogenous activity of TGases in plastids [14], [19]. These three factors could be implicated in the regulation of PSII antenna size through TGases. Ca^+2^ is a known modulator of protein functionality and, additionally, in photosynthesis it is also implicated in rapid counterion movements due to proton release [47]. GTP is also very important molecule because it could link the energy status of the cell/chloroplast (ATP) with the functionality of TGase in only a single step (conversion of ATP to GTP). Last, light could either directly or indirectly (via substrate activation) affect TGase activity optimizing regulation of the system.

Noteworthy is that the force flux plots indicate differences in CEF due to OE of TGase. Future studies for the comparison of state transition functionality in the TGZ OE could reveal interesting information.

Moreover, TGZ plants seem to store their *pmf* as electric field to a greater extent than normal. The Δψ/*pmf* of TGZ OE reached values up to 0.8 which is quite high in absolute values and about 30% higher that WT values under our experimental conditions. Usually estimates of Δψ/*pmf* in higher plants are ca 0.5 or lower [28], [48]–[50] and thus the TGZ values are quite high. The mechanism by which partitioning of thylakoid *pmf* is established and adjusted remains unclear, but *in vitro* experiments and modeling established that at least three factors are critical [24], [48], [51]: 1) the capacitance of the thylakoid membrane, which determines the Δψ generated for the transfer of a charge across the membrane; 2) the proton-buffering capacity of the lumen, which determines the relationship between translocated protons and changes in lumen pH; 3) the ionic composition of the stroma and lumen, which determines the degree to which transthylakoid counterion movements can dissipate the Δψ component.

The titer of total PAs in transformed tobacco leaves is less than half of the WT values. On the grounds that free Put can regulate partitioning of *pmf* in higher plants [24] and alter relaxation of maximal fluorescence in the dark [32] one would expect differences also in the plastidic titer of PAs between the two lines. Since free forms are substrates for the overactivated TGase, a reasonable assumption is the free titer of PAs is lower in OE. This would be consistent with the higher thylakoid associated PA titer reported in TGZ OE [20], the higher LHCII-associated PA titer in the present work and the higher relaxation rates of Fv shown in Fig. 2D. It is anticipated thylakoids with a lot of bound amines to act as a larger capacitor and increase the difference in electric potential between lumen and stroma.

Last but not least, the activation of xanthophyll cycle is about 4 times faster in TGZ OE indicating that lumen acidification occurs to a big extent (high enough absolute value of ΔpH although the ratio of ΔpH/pmf is lower than in WT). According to a qualitative comparison with a classical work in xanthophyll cycle and photoprotection [52] our data from TGZ OE resemble plants adapted in low light whereas WT resemble high light plants. These observations are not a matter of growth light conditions, because our conditions were set at 100 µmol photons m^−2^s^−1^ (which is low to medium intensity). In fact, this is due to the over-activation of TGases that, despite light signals, remodels the photosynthetic apparatus as if the light intensity was many times lower. Ignoring/circumventing light signals has detrimental effects since these plants show higher photoinhibition rates (Figures 2 and 5B) and suffer from early senescence [29].

**Figure 12:**
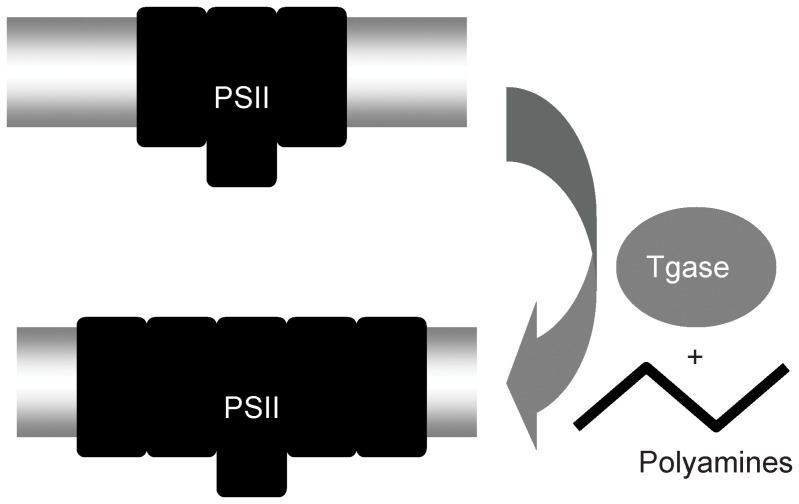
An oversimplified scheme showing a possible mode of action of TGZ in tobacco thylakoids. Higher TGase activity in chloroplast results in a higher polyamination of LHCII and CPs (this work), which in turn increase the absorption cross section of PSII. Under these conditions, antenna downregulation via qE response is getting more sensitive to LEF.

LHCII and polyamines are natural substrates of plastidal transglutaminase and TGZ action could be described by the simplified scheme in Figure 12. PSIIa units (or better PSII centers with very large absorption cross section) are accumulated due to the over expression of TGZ, which in turn increases the activity of TGase in chloroplast, and increases the polyamine content in the antenna of PSII. These findings highlight the role of polyamines and TGases in the increase of the light harvesting complex, which is important for photoadaptation.

### Concluding Remarks

In this work, firstly we prove in a recently constructed plant system that LHCII is the *in vivo* target of TGase, by co-isolating LHCII and TGase from the thylakoid membranes and showing that LHCII-bound Spd and Spm increase up to 80% in TGZ over-expressors. Secondly, we demonstrate that these post-translational modifications alter the sensitivity of antenna down regulation to the ΔpH component of proton motive force, establishing the important role of TGases in the photoprotection mechanisms and in the modulation of light harvesting through changes in antenna configuration.

## Methods

### Growth of TGZ and WT *in vitro*

As we indicated in our past work [20], tobacco plants transformed with the *tgz* gene were not viable in soil and, consequently, were *in vitro* cultured using Magenta boxes. *In vitro* culture made plant leaves more fragile and more sensitive to spectroscopic measurements. In this work we present only data from plants that responded well during measurements. The criterion was simple and photosynthesis based: only measurements that didn’t give negative values of LEF throughout the measurement protocol were included in this study. In addition, as a standard internal control WT tobacco plants grown in soil measured under similar conditions were used (data not shown).

### Spectroscopic Assays

The methods for measuring extents of qE, rates of LEF, and the relative extents of *pmf* components were as described in [46] except that a newly developed instrument was used. This instrument was based on the nonfocusing optics spectrophotometer (NoFOSpec) [53] but has been modified to allow near-simultaneous measurements of absorbance changes at four different wavelengths, by aiming four separate banks of light-emitting diodes (HLMP-CM15, Agilent Technologies, Santa Clara, CA), each filtered through a separate 5-nm bandpass interference filter (Omega Optical, Brattleboro, VT), into the entrance of a compound parabolic concentrator. The photodiode detector was protected from direct actinic light by a Schott BG-18 filter. Current from the photodiode was converted to a voltage by an operational amplifier and the resulting signal was ac-filtered to remove background signals and sampled by a 16-bit analog-to-digital converter on a personal computer data acquisition card (DAS16/16-AO, Measurement Computing, Middleboro, MA). Timing pulses were generated by digital circuitry (PC card D24/CTR 3, Measurement Computing) controlled by software developed in-house. The duration of the probe pulses was set at 10 µs. Actinic illumination was provided by a set of 2 red light-emitting diodes (HLMP-EG08-X1000, Agilent Technologies) and controlled by the timing circuitry. Measuring pulses were typically given at 1- to 20-ms intervals. Absorbance changes at only one wavelength, 520 nm, were used to estimate rapid (<1 s total trace time) changes in ECS, where its signal predominates on this timescale. For longer traces, significant contributions from light scattering have been observed [53]. To correct for this, absorbance changes of three wavelengths, 505, 520, and 535 nm, were collected. The three wavelength traces were recorded near-simultaneously, with each light-emitting diode band being pulsed in sequence at 20-ms intervals. Each complete set of three pulses was deconvoluted by using the procedure described previously [28] to obtain estimates of ECS. The instrument was also used to measure changes in chlorophyll *a* fluorescence yield by using the 520-nm light-emitting diode bank as a probe beam, as described previously [28]. Saturation pulses (>7,000 µmol of photons m^−2^ s^−1^ photosynthetically active radiation) were imposed by using light from the two red actinic LEDs, filtered through heat-absorbing glass. Actinic light was filtered out by using an RG-695 Schott glass filter. Saturation pulse-induced fluorescence yield changes were interpreted as described in [54], [55]. The qE component of NPQ was calculated from the saturation pulse-induced maximum fluorescence yields during steady-state illumination (Fm′) and 10 min (Fm′′) after switching off the actinic light [55], [56].

### *In Vivo* Measurements of Proton Flux and pmf Characteristics

This work and analysis are made possible by newly introduced techniques that allow us to noninvasively probe the ‘‘proton circuit’’ of photosynthesis. For the theoretical framework for these methods see [24], [28] and refs therein. These techniques take advantage of the ECS (sometimes called Δ520 or ΔΑ518) of certain carotenoid species that naturally occur in the thylakoid membranes. The ECS is a linear indicator of changes in transthylakoid Δψ [57] and is particularly useful for our studies because it responds to the transthylakoid movement of protons, as well as other charged species. We probed the ECS by using a previously described technique called dark-interval relaxation kinetic analysis [53], in which steady-state photosynthesis is perturbed by short (up to 0.5 s), dark intervals, allowing the photosynthetic apparatus to relax in ways that reveal information about the system in the steady state [53]. The amplitude of the light–dark ECS signal (ECS_t_) parameter was obtained by taking the total amplitude of the rapid phase of ECS decay from steady state to its quasistable level after ∼300 ms of darkness [46]. As previously discussed, ECS_t_ should reflect total light–dark *pmf* (i.e., Δψ+ΔpH) [2], [46], [51]. The dark-interval relaxation kinetic analysis technique can also reveal information about the relative conductivity of the ATP synthase to protons, a parameter termed *g*_H_^+^ [46], [51].

Because the ATP synthase is the highest conductance proton efflux pathway, decay of the ECS reflects flux through this enzyme [2]. Dark-interval relaxation kinetic analysis over longer periods of darkness can reveal information regarding the Δψ and ΔpH components of *pmf* [2], [51]. Initially, after the onset of illumination, *pmf* is stored predominantly as Δψ, because most protons are buffered and the capacitance of the membrane is relatively low. Over time, Δψ relaxes because of relatively slow movements of counterions, allowing the accumulation of free protons and subsequent buildup of ΔpH [2]. When the actinic light is rapidly shuttered, proton translocation into the lumen is rapidly halted, but proton efflux continues until *pmf* either completely collapses or comes into equilibrium with the ATP/(ADP + Pi) couple by means of the ATP synthase. Because of luminal proton buffering, Δψ will collapse more rapidly than ΔpH. Even after steady-state Δψ is dissipated, ΔpH will continue to drive proton efflux, establishing an inverse Δψ, positive on the stromal side of the thylakoid membrane. In our measurements, this inverse Δψ phase is measured as an inverted ECS signal. Under appropriate conditions [2], [51], the extent of the inverted Δψ should be proportional to the light-driven ΔpH component of *pmf*. We thus used the amplitudes of ECS kinetic components as estimates of light-driven Δψ and ΔpH.

For the deconvolution all traces were normalized to the initial dark value (i.e. before actinic) and then the following equation was used [24]: ECS_520_ =  A520 – 0.5×A535 – 0.5×A505.

### Fluorescence Measurements *in vivo*

Non-photochemical quenching of Chl fluorescence (NPQ) was determined in samples exposed to actinic light of 110 µmol photons m^−2^s^−1^ using PAM-210 Fluorometer (Heinz Walz, Germany). Samples were incubated in the dark for at least 10 min prior to measurement. The NPQ-parameter was calculated according to the equation: NPQ =  (F_M_ – F_M_′)/F_M_′ [52].

For the fluorescence induction measurements, the portable Plant Efficiency Analyzer, PEA (Hansatech Instruments) was used as previously described [34]. The method is based on the measurement of a fast fluorescent transient with a 10 µs resolution in a time span of 40 µs to 1s. Fluorescence was measured at a 12 bit resolution and excited by three light-emitting diodes providing an intensity of 3000 µmol photons m^−2^s^−1^ of red light (650 nm). For the estimation of Ka, DCMU inhibited electron transport after Q_A_ and analysis of the area closure was performed according to the method of Melis as modified in [20].

### Isolation of Thylakoid Membranes

Thylakoids were isolated as previously described [20], [26].

### Isolation of Light Harvesting Antenna

For the isolation of LHCII proteins, the solubilised thylakoid preparations were subjected to ultracentrifugation on a continuous sucrose gradient as previously described [33], [34]. The concentration of Chl in the thylakoid sample loaded on the 5–22% continuous sucrose gradients was always adjusted to a Chl concentration of 600 µg/ml. Up to this concentration no sediment could be detected at the bottom of the gradients. Ultracentrifugation was performed at 170,000×g (42000 rpm, rotor SW-40, Beckman L8–80M ultracentrifuge) for 18 h at 4°C. After separation, both the monomer and oligomer fraction [26] was used for spectroscopy studies in a 15 mM tricine buffer (pH 7), containing 0.15 M Sucrose. Usually the Chla/b ratio of the fraction used was around 1.4±0.15.

The model of LHCII (monomer) was performed by using the software The PyMOL Molecular Graphics System, Version 1.3, Schrödinger, LLC (http://www.pymol.org/citing), based on the file 2BHW opened by Protein Data Bank (URL:http://www.rcsb.org/pdb/) for LHCII [58] and 3PL9 for CP29 [59].

### Western Blotting

Western immunoblotting was performed according to Towbin method [60]. Isolated LHCII protein extracts (100 µg) from transformed and untransformed leaves were loaded per well and electrophoresed in a 10% polyacrylamide gel. Proteins were separated by SDS-PAGE according to Laemmli [61] in a Mini-Protean III system (Bio-Rad, Hercules, CA, USA) and further were transferred to a nitrocellulose membrane (GE Healthcare, Little Chalfont, UK) on wet systems (Bio-Rad, Hercules, CA, USA) according to manufacturer’s instructions. For immunoblotting, membrane blocking was performed with non-fat dry milk (5 or 10%, w/v) in PBS and washed in PBS with Tween 20 (0.1 to 0.3%, v/v). To detect TGase presence, a polyclonal antibody raised against generic plant transglutaminase (AbH) [10] and a specific antibody raised against the C-terminal TGZ sequence (Abpep) [16] were used as primary antibodies at 1∶1000 and 1∶500 dilution, respectively. For Lhcb1–6 detection, the respective specific primary antibodies (Agrisera) were used, at 1∶5000 dilution. A peroxidase-conjugated goat anti-rabbit IgG (Sigma-Aldrich, Spain A0545) was used in all cases as secondary antibody, at 1∶15000 dilution, except in the case of AbH, where a peroxidase-conjugated donkey anti-chicken IgY (Jackson Immunoresearch Lab. Inc, Pennsylvania, USA) diluted 1∶6700 was used as secondary antibody. Detection was performed using the ECL western blotting detection system (GE Healthcare, Little Chalfont, UK).

### Polyamine Analysis by High Performance Liquid Chromatography (HPLC)

Polyamines were extracted as previously described and analysed following the method of Kotzabasis [62]. Briefly, for polyamine analysis leaf powder after liquid nitrogen was suspended in 1 N NaOH. A volume of 0.2 ml from the hydrolysate was mixed with 36% HCl in a ratio of 1∶1 (v/v) and incubated at 110°C for 18 h. The hydrolysate was evaporated at 70–80°C. The dried products were re-dissolved in 0.2 ml of 5% (v/v) perchloric acid. To identify and estimate the polyamines, the samples were derivatized by benzoylation, as is previously described [34]. For this purpose, 1 ml of 2 N NaOH and 10 µl benzoylchloride were added to 0.2 ml of the hydrolysate and the mixture vortexed for 30 s. After 20 min incubation at room temperature, 2 ml of saturated NaCl solution were added to stop the reaction. The benzoylpolyamines were extracted three times into 2–3 ml diethylether; all ether phases collected and evaporated to dryness. The remaining benzoylpolyamines were re-dissolved in 0.2 ml of 63% (v/v) methanol and 20 µl aliquots of this solution were injected into the HPLC system for the polyamine analysis, as described previously [34]. The analyses were performed with a Shimadzu Liquid Chromatography apparatus (LC-10AD) equipped with a SPD-M10A diode array detector (Shimadzu SPD-M10A) and a narrow-bore column (C18, 2.1×200 mm, 5 µm particle size Hypersyl, Hewlett-Packard, USA).
